# Membranous nephropathy in patients with HIV: a report of 11 cases

**DOI:** 10.1186/s12882-020-02042-x

**Published:** 2020-09-18

**Authors:** Vivek Charu, Nicole Andeen, Vighnesh Walavalkar, Jessica Lapasia, Jin-Yon Kim, Andrew Lin, Richard Sibley, John Higgins, Megan Troxell, Neeraja Kambham

**Affiliations:** 1 Department of Pathology, Stanford University School of Medicine, 300 Pasteur Drive, H2110A, Stanford, CA 94304 USA; 2 Department of Pathology, Oregon Health & Science University, Portland, OR USA; 3 Department of Pathology, University of California at San Francisco, San Francisco, CA USA; 4 Department of Nephrology, The Permanente Medical Group, San Francisco, CA USA; 5 Department of Nephrology, The Permanente Medical Group, Sacramento, CA USA

## Abstract

**Background:**

Membranous nephropathy (MN) has been recognized to occur in patients with human immunodeficiency virus (HIV) infection since the beginning of the HIV epidemic. The prevalence of phospholipase A2 receptor (PLA2R)-associated MN in this group has not been well studied.

**Methods:**

We conducted a retrospective review of electronic pathology databases at three institutions to identify patients with MN and known HIV at the time of renal biopsy. Patients with comorbidities and coinfections known to be independently associated with MN were excluded.

**Results:**

We identified 11 HIV-positive patients with biopsy-confirmed MN meeting inclusion and exclusion criteria. Patient ages ranged from 39 to 66 years old, and 10 of 11 patients (91%) were male. The majority of patients presented with nephrotic-range proteinuria, were on anti-retroviral therapy at the time of biopsy and had low or undetectable HIV viral loads. Biopsies from 5 of 10 (50%) patients demonstrated capillary wall staining for PLA2R. Measurement of serum anti-PLA2R antibodies was performed in three patients, one of whom had positive anti-PLA2R antibody titers. Follow-up data was available on 10 of 11 patients (median length of follow-up: 44 months; range: 4–145 months). All patients were maintained on anti-retroviral therapy (ARV) and 5 patients (52%) received concomitant immunosuppressive regimens. Three patients developed end-stage renal disease (ESRD) during the follow-up period.

**Conclusions:**

MN in the setting of HIV is often identified in the setting of an undetectable viral loads, and similar to other chronic viral infection-associated MNs, ~ 50% of cases demonstrate tissue reactivity with PLA2R antigen, which may be seen without corresponding anti-PLA2R serum antibodies.

## Background

The spectrum of renal pathology in patients infected with HIV is broad and includes HIV-associated nephropathy (HIVAN), focal and segmental glomerulosclerosis (FSGS), thrombotic microangiopathy, and HIV-associated immune complex kidney disease (HIVICK), among others [1, 2]. HIVICK is a heterogenous category of disease, comprised of specific, well-characterized glomerular diseases (e.g. IgA nephropathy, membranoproliferative glomerulonephritis, membranous nephropathy etc.), as well as immune-complex mediated diseases, not otherwise specified, including those with “lupus-like” features [3, 4]. In the era of antiretroviral therapy, some biopsy series in patients infected with HIV have suggested that the prevalence of HIVICK exceeds that of HIVAN [5]. The precise role that HIV plays in the development of HIVICK remains unknown, and studies of clinical outcomes in HIVICK have been hampered largely by small sample sizes and the heterogeneity of diseases falling into this umbrella category [6, 7].

Among patients with HIVICK, membranous nephropathy (MN) has been reported in ~ 3–30% of cases [7, 8]. Several studies have demonstrated that patients with HIV and MN are often co-infected with hepatitis C (HCV), hepatitis B viruses (HBV), and/or syphilis which are themselves independently associated with MN [5, 9–12], challenging a causal association between MN and HIV. To date, no study specifically investigating MN in patients with HIV who lack known co-infections has been reported. In the past decade, many important advances in the understanding of idiopathic MN have been made, including the discovery of antibodies to the M-type phospholipase receptor (PLA2R) antigen [13]. The prevalence and role of PLA2R antibodies in MN in patients with HIV has thus far not been studied. Here we report on the biopsy findings and clinical outcomes of 11 patients with HIV and MN without known co-infections.

## Methods

### Study design

We conducted a retrospective review of the electronic pathology databases at Stanford University School of Medicine, Oregon Health & Science University School of Medicine, and the University of California San Francisco School of Medicine. Where possible, the electronic medical record was used to provide clinical and laboratory data. This study was approved by the institutional review boards at all three medical centers.

### Inclusion criteria

Native kidney biopsies occurring between June 1, 2000 and June 1, 2019, with diagnoses of MN among patients with known HIV infection at the time of biopsy.

### Exclusion criteria

Patients with co-existing HCV, HBV, syphilis or other infections reported in the setting of MN either at the time of biopsy or on subsequent follow-up were excluded. Patients with known connective tissue disorders or on medications known to be associated with MN were also excluded.

### Biopsy processing

Standard processing of kidney biopsies at all three institutions include light microscopy, immunofluorescence, and electron microscopy. For light microscopy, biopsy specimens were stained with hematoxylin-eosin, periodic acid-Schiff, Jones methenamine silver, and in some cases, Masson trichrome. For immunofluorescence, cryostat sections were stained with polyclonal fluorescein isothiocyanate (FITC)-conjugated antibodies to IgG, IgM, IgA, C3, C1q, kappa and lambda light chains, fibrinogen, and albumin, as per routine clinical testing. Electron microscopy was performed as per clinical routine. For cases in which adequate frozen tissue for immunofluorescence was available, additional staining for IgG antibody subclasses (IgG1, IgG2, IgG3 and IgG4), as well as PLA2R was performed. Air dried cryostat sections were incubated with PLA2R antibody (Sigma-Aldrich HPA012657, 1:5 dilution) for 60 min, followed by FITC labeled secondary antibody (Vector, FI-1000, in PBS buffered diluent) for 60 min with PBS rinse in between and after. Sections were cover slipped with Dako Fluorescent Mounting media. Staining was performed using appropriate positive and negative controls.

For study purposes, immunoperoxidase staining for PLA2R was performed at Stanford. The deparaffinized formalin-fixed sections were subjected to antigen retrieval using citrate (pH 6), followed by incubation in 1% H202 for 10 min and normal serum blocking (1:40) for 30 min. The sections were incubated with PLA2R antibody (Sigma -Aldrich, HPA012657) for 70 min (dilution 1:6000) and HRP (Vector MP-7401 anti-Rabbit IgG) reagent for 30 min with PBS washes in between. 3,3′-Diaminobenzidine (DAB) Liquid Substrate System was used followed by counterstaining with hematoxylin. Discrete granular capillary wall staining was considered positive. Absent staining, or non-discrete cytoplasmic staining was considered negative.

## Results

### Patient demographics and clinical characteristics at the time of renal biopsy

We identified 11 HIV-positive patients with biopsy-confirmed MN meeting our inclusion and exclusion criteria. Patient demographics and clinical data at the time of biopsy are presented in Table 1. Patient ages ranged from 39 to 66 years old, and 10 of 11 patients (91%) were male. The most common presentation was nephrotic-range proteinuria (median 24-h urine protein 9 g; median urine protein-to-creatinine ratio [UPCR] 3.9 g/g), and four patients presented with concomitant acute kidney injury (defined by an increase in serum creatinine of > 1.5-fold from known baseline). The majority of patients had low or undetectable HIV viral loads (< 75 copies/mL; 89%), and 90% were on anti-retroviral (ARV) therapy at the time of biopsy. Three patients had coexisting type II diabetes mellitus and 3 patients had a history of hypothyroidism. 1 patient was dialysis-dependent at the time of biopsy (Patient 4).

**Table 1:** Clinical and laboratory data at the time of renal biopsy in 11 patients with HIV and membranous nephropathy

Patient	Sex	Age	Race/Ethnicity	HIV-1 RNA (copies/mL)	CD4 (count/mL)	SCr	Dialysis*	ARV	Urine protein (g/24 h)	UPCR (g/g)	Albumin (g/dL)	Other medical conditions
1	M	54	AA	< 75	983	1.3	N	Y	7.0	16.5	2.1	HTN; Hypothyroidism
2	M	61	AA	931	331	3.3	N	N	10.6	4.2	3.3	Diabetes; HTN; aFib
3	M	42	Hisp.	–	–	1.04	N	Y	–	–	2.1	–
4	M	59	AA	39	419	16.9	Y	Y	–	1.9	4.0	HTN; CVA; HLD
5	M	39	AA	< 48	718	0.77	N	Y	13.0	8.16	1.6	Obesity; HLD
6	M	62		< 48	375	2.23	N	Y	–	3.86	2.8	Diabetes
7	F	69	Cauc.	< 48	296	1.10	N	Y	4.0	10.2	3.0	Diabetes, Asthma; aFib; COPD; CHF
8	M	63	–	“low”	1073	“normal”	N	Y	“heavy”	–	–	Hypothyroidism
9	M	66	–	Undetectable		3.2	N	Y	8.0	–	–	–
10	M	66	–	Undetectable	350	1.0	N	Y	10.0	–	–	Hypothyroidism
11	M	55	Pac. Isl.	< 75	234	1.16	N	Y	3.0	2.9	2.0	–

Abbreviations: M: male; F: female; AA: African-American; Hisp.: Hispanic; Cauc.: Caucasian; Pac. Isl.: Pacific Islander; SCr: serum creatinine; ARV: antiretroviral therapy; UPCR: urine protein to creatinine ratio; HTN: hypertension; aFib: atrial fibrillation; CVA: cerebrovascular accident; HLD: hyperlipidemia; COPD: chronic obstructive pulmonary disease; CHF: congestive heart failure.*Refers to dialysis-dependence at the time of biopsy

### Renal biopsy findings

#### Light microscopy

A summary of light microscopic biopsy findings is provided in Table 2. The average number of glomeruli sampled was 31 (range: 7–46), with an average of 15% global sclerosis (range: 0–43%). The degree of interstitial fibrosis and tubular atrophy ranged from mild (*n* = 7) to moderate (*n* = 2) to severe (n = 2). No biopsy specimen contained crescents or glomerular necrosis.

**Table 2:** Light (LM) and electron microscopic (EM) findings in renal biopsies in 11 patients with HIV and membranous nephropathy

Patient	Diagnoses	Total glomeruli*	GS (%)	IFTA (%)	FSGS	Arteriosclerosis	EM Deposit location	EM MN stage	FPE (%)
1	MN	7	0	< 5	0	Normal/minimal	NA	NA	NA
2	HIVAN; MN; mild DN	42	43	90	Collapsing	Severe	Mes; SEpi (segmental); IM	II-III	60–70 (variable)
3	MN; TIN	38	5	10	0	Mild	SEpi; IM	II-IV	100
4	MN; TIN	39	3		0	Moderate	Mes; SEpi; IM	II-III	50 (variable)
5	MN	32	9	5	0	Mild	SEpi	I-II	100
6	MN	46	42	75	NOS	Severe	SEpi; IM	II-III	100
7	MN	25	12	10	0	Mild	SEpi	I-II	100
8	MN	28	0	5	0	Moderate	SEpi	II	100
9	MN	14	36	50	0	Moderate	SEpi	III	100
10	MN; LLN	28	0	10	0	Mild	Mes (rare); SEndo (rare); SEpi (segmental)	I	60–70 (variable)
11	MN	45	13	30	NOS	Severe	Mes; SEpi	IV	80%

Abbreviations: GS: glomerulosclerosis; IFTA: interstitial fibrosis and tubular atrophy; FSGS: focal and segmental glomerulosclerosis; FPE: foot process effacement; MN: membranous nephropathy; TIN: tubulointerstitial nephritis; LLN: lupus-like nephropathy; DN: diabetic nephropathy; Mes.: mesangial; SEpi.: subepithelial (diffuse unless otherwise specified); SEndo.: subendothelial; IM: intramembranous. Deposits are considered diffuse unless otherwise specified. *Describes total glomeruli sampled for LM, EM, and IF

**Fig. 1:**
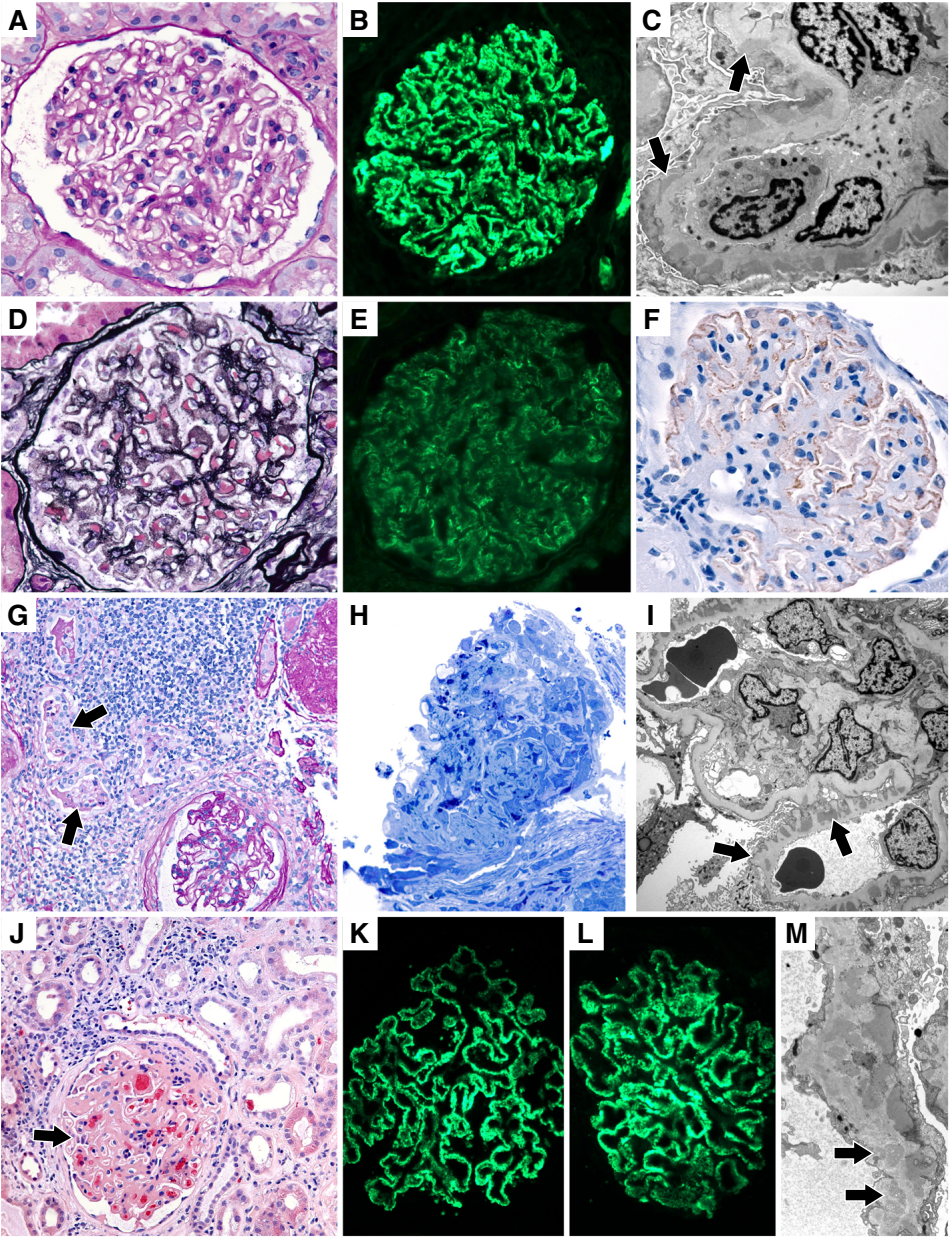
Biopsy Findings in patients with HIV infection and membranous nephropathy (MN). **A-C:** Early MN A) Well preserved glomerulus with mildly thickened basement membranes (PAS, × 400). B) Diffuse granular capillary wall staining with IgG. C) Subepithelial electron dense deposits (arrows) with associated basement membrane spikes (× 10,000; Patient 5). **D-F:** Chronic MN with extensive tubular atrophy and hypertensive arteriosclerosis. D) Glomerulus with basement membrane spikes (JMS, × 400). E) Diffuse granular capillary wall staining with PLA2R immunofluorescence. F) Discrete capillary wall deposits with PLA2R immunohistochemistry. (Patient 6). **G-I:** MN with co-existent HIVAN-like features. G) Prominent interstitial inflammation and intratubular neutrophils (arrows) in a biopsy with segmental MN (not shown) (PAS, × 200). H) Glomerulus with epithelial cell proliferation and segmental capillary wall collapse (Toluidine-Blue, × 400). I) Segmental subepithelial and intramembranous electron dense deposits (arrows). Mesangial sclerosis attributed to co-existent early diabetic nephropathy. Focal mesangial deposits were also present (not shown) (× 2700; Patient 2). **J-M:** MN with chronic active tubulointerstitial nephritis. J) Interstitial edema and inflammation associated with tubular injury. Glomerulus has thickened basement membranes (arrow) (H&E, × 200). K) Diffuse granular capillary wall immunofluorescence staining with IgG4 subclass. L: Similar staining with PLA2R immunofluorescence. M) Intramembranous deposits with electron lucent areas (arrows) (stage III-IV MN deposits; Patient 3)

Three cases demonstrated evidence of focal and segmental sclerosis (FSGS). One biopsy demonstrated FSGS with collapsing features in addition to MN (Patient 2; Fig. 1); this patient also had elevated HIV-1 viral load, raising the possibility of concomitant HIV-associated nephropathy (HIVAN). Other light microscopic features of HIVAN, including microcystic tubular dilatation, were not seen, although the extensive interstitial fibrosis and tubular atrophy (> 90% of the cortex sampled) precluded accurate assessment (Fig. 1).

Two biopsies demonstrated evidence of concomitant chronic active tubulointerstitial nephritis, characterized by interstitial inflammation, tubulitis and focal interstitial eosinophils (Patients 3 and 4; Fig. 1). One patient (Patient 4) presented with acute renal failure requiring dialysis, with the renal biopsy demonstrating interstitial nephritis without tubular crystals, which was attributed to Tenofovir (subsequently discontinued). Overall, the clinical presentation of fulminant renal failure with tubulointerstitial nephritis in this patient suggested a pre-existing MN unrelated to the acute presentation. In both cases, the histologic features (in conjunction with the lack of prominent plasma cell infiltrate) did not support a diagnosis of IgG4-related tubulointerstitial nephritis.

#### Immunofluorescence microscopy findings

**Fig. 2:**
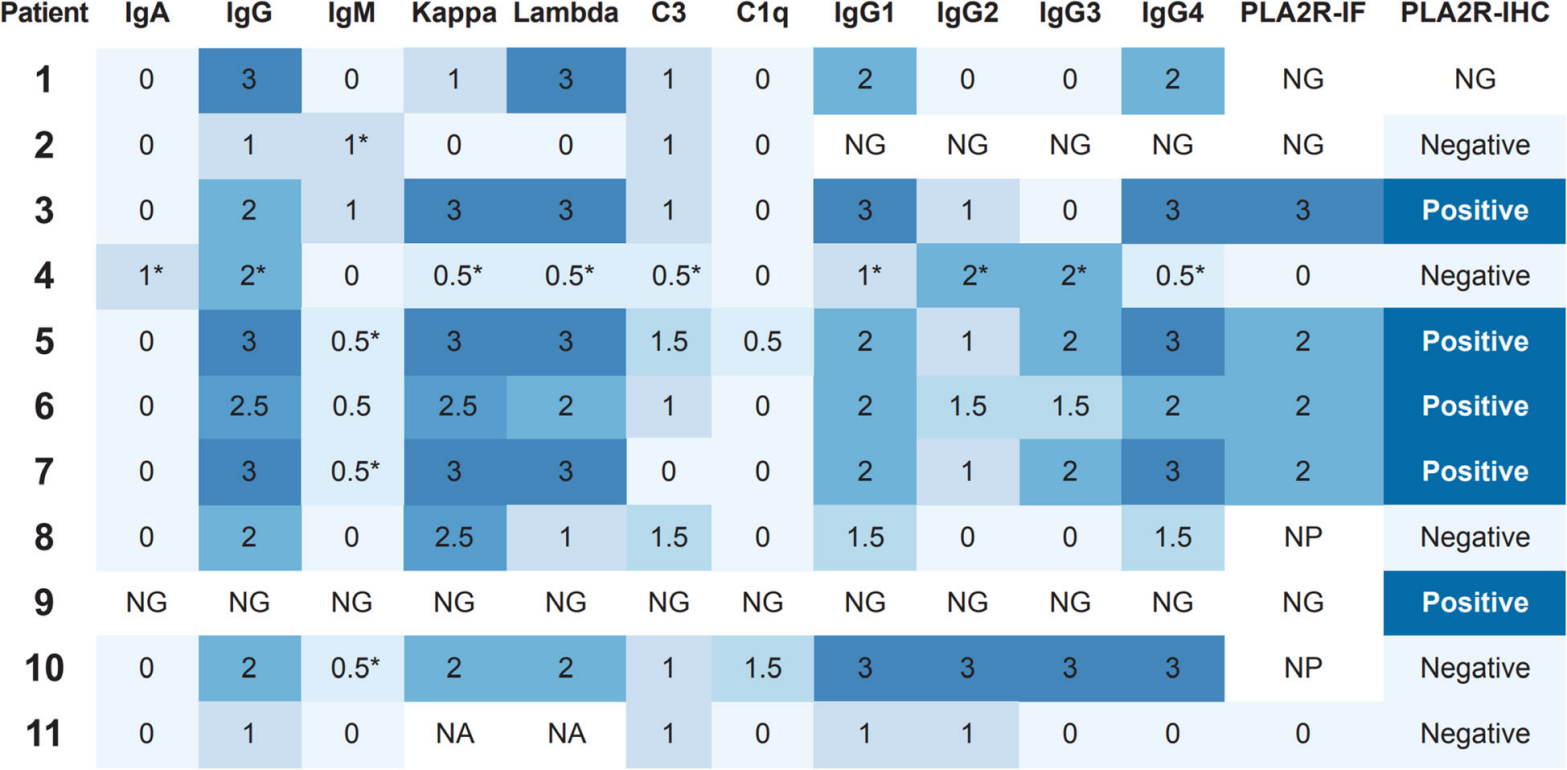
Immunofluorescence microscopy findings in 11 patients with HIV and MN. Intensity of staining was graded on a scale of 0–3, and color coded in the figure from light blue (0) to dark blue (3). A value of 0.5 refers to “trace” staining. All numbers reflect the intensity of granular capillary wall staining, unless otherwise specified. *Refers to granular mesangial and segmental capillary wall staining. Abbreviations: NG: No glomeruli present for evaluation; NP: Not performed

Tissue submitted for immunofluorescence microscopy was adequate for evaluation in 10 cases (91%; Fig. 2). Granular capillary wall staining for IgG, kappa and lambda light chains was seen in all cases (10/10; 100%), with granular capillary wall staining for C3 present in 9 of 10 cases. Faint (trace to 1+) granular mesangial staining for IgA and kappa/lambda light chains was seen in one case (Patient 4); weak granular mesangial staining for IgM (trace-1+) was seen in 5 cases (50%). One case (Patient 10) demonstrated quasi full-house granular staining along capillary loops (IgG, C3, C1q, kappa and lambda). In conjunction with the rare mesangial and subendothelial deposits identified by electron microscopy, this MN case had “lupus-like” features [4].

#### PLA2R and IgG subclass staining

Phospholipase A2 Receptor (PLA2R) staining by immunohistochemistry on paraffin embedded tissue or immunofluorescence on frozen tissue was performed on all cases, 10 of which had glomeruli present for evaluation (91%; Fig. 2). Immunofluorescence for IgG subclasses was performed in 9 cases (82%). 5 of 10 cases demonstrated capillary wall staining for PLA2R (50%); among cases in which PLA2R staining was performed by both immunofluorescence and immunohistochemistry, there was 100% concordance between the two methods. For cases in which IgG subclass staining was performed, cases that were PLA2R-positive demonstrated IgG4-dominant or co-dominant staining. Cases with dominant or co-dominant staining for IgG2 and/or IgG3 were PLA2R-negative (Fig. 2). Notably, all cases with mesangial electron dense deposits seen by electron microscopy (4/4) were PLA2R-negative (Tables 2 and 3).

**Table 3:** Clinical outcomes after diagnosis of membranous nephropathy

No.	Treatment	FU (months)	Renal outcome	Last CD4	Last HIV*	Last sCr	Last UPCR (g/g)**	Serum anti-PLA2R***
1	ACEi/ARB; MMF; Pred; ARV then MMF switched to CYC within 1 yr	145	ESRD/Dialysis	806	Undet.	4.4	8.4	NP
2	ACEi/ARB; MMF; Pred; ARV	140	ESRD/Dialysis; Transplant	599	Undet.	7.2	7.2	NP
3	–	81	Non-ESRD	161	Undet.	0.95	Undet.	NP
4	Discontinuation of Tenofovir	54	Non-ESRD	216	2190	1.27	–	NP
5	ACEi/ARB; PredTac; then Ritux; mPred	9	Non-ESRD	971	Undet.	1.16	11.9	153 RU/mL
6	ACEi/ARB; Pred; then CYC added	9	Non-ESRD	244	Undet.	2.73	2.02	< 1:10
7	ACEi/ARB	4	Non-ESRD	243	Undet.	1.9	1.0	< 1:10
8	–	114	Non-ESRD	–	–	0.89	–	NP
9	ACEi/ARB;Pred;CYC	20	ESRD/dialysis	–	Undet.	–	–	NP
10	ACEi/ARB	42	Non-ESRD	–	Undet.	“normal”	Undet.	NP
11	ACEi/ARB	44	Non-ESRD	1117	Undet.	2.8	0.25	NP

Abbreviations: FU: Follow-up; sCr: serum creatinine; UPCR: urine protein-to-creatinine ratio; MMF: mycophenolate mofetil; Pred: prednisone; ARV: antiretroviral therapy; ACEi/ARB: Angiotensin converting enzyme inhibitor/angiotensin receptor blocker; Tac: Tacrolimus; Ritux: Rituximab; mPred: methylprednisolone; CYC: cyclophosphamide; Undet.: undetectable; NP: Not performed.*Undet. refers to undetectable HIV-1 RNA levels, the lower limit of detection being < 75 copies/mL.**Undet. refers to undetectable urine protein by dipstick analysis. *** < 1:10 refers to a negative titer of serum anti-PLA2R antibodies

Measurement of serum anti-PLA2R antibodies was performed in three patients whose biopsies demonstrated tissue positivity for PLA2R by immunofluorescence/immunohistochemistry (Patients 5–7; Table 3). Only one such patient demonstrated positive anti-PLA2R antibody titers (Patient 5; Table 3).

#### Electron microscopic findings

Tissue submitted for electron microscopy was adequate for evaluation in 10 cases (91%). All cases demonstrated subepithelial deposits diagnostic of membranous nephropathy, with the majority of biopsies demonstrating diffuse subepithelial deposits (80%), and the remainder demonstrating segmental deposits (Table 2). Rare mesangial deposits were identified in 4 cases (40%), and rare subendothelial deposits were seen in one case (Patient 10). In one case, a microtubular substructure to the subepithelial deposits was present (Patient 11). The extent of foot process effacement ranged from 50 to 100% (Table 2). Tubulo-reticular inclusions were not identified in any case.

#### Treatment approaches and clinical outcomes

Follow-up data was available on 10 of 11 patients (91%; median length of follow-up: 44 months; range: 4–145 months; Table 3), with specific information on treatment approaches in 9 patients. All patients were maintained on anti-retroviral therapy (ARV). 5 patients (52%) received concomitant immunosuppressive regimens, including mycophenolate mofetil, prednisone, tacrolimus, cyclophosphamide or rituximab (Table 3). Among patients receiving concomitant immunosuppression, HIV viral loads remained low/undetectable, and CD4 counts were within normal limits on follow-up (where data were available).

One patient (Patient 4) who presented with severe acute kidney injury had biopsy findings of MN and chronic active tubulointerstitial nephritis and was treated with angiotensin blockade and discontinuation of tenofovir, without concomitant immunosuppression. This patient underwent a remarkable recovery in renal function (Table 3).

One patient (Patient 5) had serial serum anti-PLA2R autoantibody measurements, demonstrating a reduction in anti-PLA2R titers from 153 RU/mL to 69 RU/mL within ~ 6 months; this patient was treated with an immunosuppressive regimen that included rituximab. Despite the reduction in serum anti-PLA2R titers, this patient had residual nephrotic-range proteinuria during the follow-up period (Table 3).

Three patients developed end-stage renal disease requiring dialysis, and one patient underwent renal transplant during the follow-up period (Table 3).

## Discussion

Though MN has been recognized in association with HIV since the beginning of the HIV epidemic [14, 15], the precise causal relationship between HIV and MN remains unclear, and establishing a causal link between HIV and MN is challenging. Patients with HIV are at increased risk for being coinfected with HBV, HCV and syphilis, all of which are independently associated with MN. Here we report clinical characteristics, renal biopsy findings (including tissue PLA2R staining), treatment approaches and renal outcomes in 11 patients with HIV and MN who lack comorbidities known to be independently associated with the development of MN.

The proposed mechanisms of renal injury in HIV infection vary by disease entity [16]. For example, HIVAN occurs primarily in patients with active HIV infection and HIV RNA has been localized within podocytes and tubular epithelial cells, implicating direct viral infection in disease pathogenesis [16, 17]. In the setting of HIV-associated thrombotic microangiopathy, HIV virions and peptides have been shown to induce endothelial cell apoptosis and have prothrombotic effects in vivo [16]. In contrast, pathogenic mechanisms driving HIVICK, including MN, remain unclear, in part due to the heterogeneity of diseases within this group and the variety of clinical presentations encountered. One hypothesis is that, in the setting of active HIV replication and a functional immune system, polyclonal antibodies against HIV epitopes leads to glomerular deposition of circulating immune complexes. Some such evidence exists for “HIV-associated” IgA nephropathy, in which circulating immune complexes of HIV p24 and gp120 antigens and IgA were identified in the presence of active HIV infection [18]. However, antibody responses in the setting of high HIV antigen burden cannot account for all presentations of HIVICK, as HIVICK is often encountered in patients with undetectable viral loads [7], indicating that a dysregulated host immune system may also play an important role.

In the specific scenario of MN in the setting of HIV, few studies have explored the precise mechanisms of disease, but a review of cases reported in the literature sheds some light. An early study of HIV-associated renal disease demonstrated the presence of the HIV genome in renal biopsy tissue across a wide spectrum of renal lesions, including one case of MN (in a patient with concomitant HBV) [18]. One patient with HIV-associated MN who lacked other co-infections demonstrated remission of proteinuria with antiretroviral therapy and angiotensin-receptor blockade [19]. Taken together, these findings lend weak support for the hypothesis that HIV replication is associated with the development of MN. In contrast, most other studies have demonstrated that patients with MN in the setting of HIV have well-controlled HIV viral loads, with mean CD4 counts greater than 200 cells/ul [7, 20]. Another case report of MN in the setting of HIV infection demonstrated response to prednisone [21], suggestive of host (auto) immune mechanisms playing a role in pathogenesis. In line with these latter studies, we note that 89% of patients in our study had low or undetectable viral loads, and fully half were treated with immunosuppressive regimens in addition to antiretroviral therapy (and/or angiotensin receptor blockade). In the context of studies reported in the literature, our data would support a role for host immune system dysregulation in the development of MN in patients with HIV, as opposed to active HIV replication.

Our study is the second to systematically report tissue PLA2R antibody status in MN among patients with HIV [22]. Five of ten cases tested (50%) demonstrated positive PLA2R staining in the glomerular capillary walls (5/10). Evaluation of IgG subclasses (IgG1-G4) revealed that these PLA2R+ membranous deposits were IgG4 dominant (or codominant), as has been previously described in primary/idiopathic MN [23]. Although serum data was only available in three patients, all with tissue-positivity for PLA2R, only one patient had elevated serum anti-PLA2R antibodies at the time of testing. A recent study reported on 15 HIV-positive patients with MN, of whom 3 had concomitant HCV; among 12 patients with available information on tissue PLA2R staining, 7 tested positive (58%) [22]. To our knowledge, only two other cases of HIV-associated MN in which tissue PLA2R staining was performed are reported in the literature, one of which was negative [24]. The second reported case was PLA2R positive (tissue stain), in a patient with concomitant HCV infection [25].

Though the chance occurence of primary/idiopathic PLA2R-associated MN independent of HIV infection cannot be excluded, our data, in the context of other studies, may suggest an associated between the development of anti-PLA2R autoantibodies and HIV. Indeed, numerous autoimmune diseases affecting a variety of organs have been linked to viral infections [26]. Hypothesized mechanisms include: (1) molecular mimicry, in which similarities between viral peptides and self-antigens trigger a virus-directed cross-reactive response [27], and (2) “bystander effect”, in which tissue damage results due to over-reactive antiviral immune responses, with subsequent release of self-antigens which may further perpetuate autoimmune-mediated injury via epitope spreading [28, 29]. Recent work has identified a major epitope, the N-terminal cysteine-rich ricin domain of PLA2R, that is recognized by 90% of human anti-PLA2R autoantibodies [30]. The linear sequence of 31 peptides identified as the major epitope targeted by human anti-PLA2R autoantibodies shares no sequence homology with HIV, HBV or HCV peptides, lending no definite support for molecular mimicry as a cause of anti-PLA2R antibody formation (data not shown). Recent studies have demonstrated that ~ 64% of MN cases in the setting of HBV [31] and ~ 64% in the setting of HCV demonstrate tissue-positivity for PLA2R [24]. Although the mechanisms are unclear, our data raise the possibility that HIV, similar to HBV and HCV, may induce autoantibodies to intrinsic glomerular antigens, such as PLA2R, perhaps in the setting of “bystander effect” [26, 32]. Whether these viral-associated tissue-PLA2R positive cases of MN truly represent primary/idiopathic disease remains to be elucidated.

## Conclusion

In summary, MN in the setting of HIV is often identified in the setting of an undetectable viral loads, and similar to other chronic viral infection-associated MNs, ~ 50% of cases demonstrate tissue reactivity with PLA2R antigen, which can be seen without corresponding anti-PLA2R serum antibodies. These observations refine our understanding of a specific disease entity previously grouped under HIVICK, and expand the clinical and pathologic description of MN in HIV-positive patients who lack other co-infections or co-morbidities.

## Data Availability

All data generated or analysed during this study are included in this published article.

## Abbreviations

MN: Membranous nephropathy
HIV: Human immunodeficiency virus
HBV: Hepatitis B virus
HCV: Hepatitis C virus
PLA2R: Phospholipase A2 receptor
